# Influence of social media on cosmetic and gynecologic aesthetic decisions among women in Saudi Arabia

**DOI:** 10.3389/fdgth.2026.1775085

**Published:** 2026-04-07

**Authors:** Dana Sawan

**Affiliations:** Obstetrics and Gynecology Department, Faculty of Medicine, King Abdulaziz University, Jeddah, Saudi Arabia

**Keywords:** aesthetic decision-making, body image, cosmetic surgery, Saudi Arabia, social media

## Abstract

**Objective:**

To explore how social media influences cosmetic and gynecologic aesthetic decision-making among women in Saudi Arabia, with attention to behavioral, psychological, ethical, and cultural dimensions.

**Methods:**

A structured narrative review guided by SANRA principles was conducted using publications from 2018 to 2024 identified through PubMed, OpenAlex, and institutional repositories. Studies were screened thematically and synthesized using a narrative thematic approach. Studies were screened for relevance to social-media influence on cosmetic behavior, psychological motivation, professional ethics, and female genital cosmetic surgery. Thirteen studies meeting inclusion criteria were included and synthesized across four thematic domains.

**Results:**

Across Saudi and international studies, high exposure to visual social-media platforms—particularly influencer content, filters, and before-and-after imagery—was consistently associated with increased interest in cosmetic procedures. Psychological mechanisms included social comparison, appearance dissatisfaction, and desire for peer validation. Ethical concerns highlighted promotion-driven content, selective imagery, and limited risk disclosure by practitioners. In the Saudi context, cultural norms, digital engagement, and evolving social expectations interacted to shape women's motivations, including those considering gynecologic aesthetic procedures.

**Conclusions:**

Social media plays a substantial role in shaping perceptions of beauty, motivations for cosmetic and gynecologic procedures, and expectations of treatment outcomes. Clinicians should address social-media influence during counseling, promote realistic expectations, and uphold ethical communication standards. Further longitudinal and culturally grounded research is needed to clarify how digital exposure shapes women's decisions over time.

## Introduction

Over the past generation, the meaning of beauty has quietly evolved from an aesthetic preference into a social language—one that communicates identity, confidence, and belonging. In the modern world where beauty is derived by images, people do not merely look at themselves in the mirror, instead they compare, curate, and broadcast those reflections across digital platforms. Social media has taken the place of the mirror—reflecting not only how people look, but how they wish to be seen. Recently, cosmetic medicine has expanded quickly since before it was about treating physical flaws and now it's about helping people express themselves and feel more confident (1).

Nowhere is this transformation more evident than in societies where cultural traditions and modern technology are reshaping one another. In Saudi Arabia, where social modernization coincides with near-universal smartphone use and a young, digitally active population, social media has become an influential force in shaping ideals of beauty and self-presentation (2). The desires for aesthetic change used to be private but now we see them openly across social media through filters, hashtags, and influencer imagery. Cosmetic enhancement, previously regarded as a privilege or a taboo, has entered the mainstream as a familiar expression of confidence and well-being (3).

Across the world, visual platforms such as Instagram, Snapchat, and TikTok have created a continuous stream of idealized imagery that redefines what is perceived as “normal” or “beautiful.” These platforms do not merely display beauty—they construct it. A cross-sectional study conducted in 2018 among Saudi women found that nearly half of participants believed social media directly influenced their decision to consider or undergo a cosmetic procedure (4). Comparable international findings show that repeated exposure to aesthetic content, including before-and-after photos and influencer testimonials, increases the desire for elective enhancement (5). This shows that the digital media has become a silent architect of beauty standards, framing cosmetic intervention as a routine response to social visibility.

The psychological effects of this trend are powerful. Many people now compare themselves to the flawless images they see online, sometimes without realizing how much those comparisons affect them (6). Many people view cosmetic procedures as a way to boost their confidence, where these choices are usually shaped by social pressures and the ongoing need for approval through likes and comments. Even efforts such as “body positivity,” though intended to encourage acceptance, often keep appearance at the center of attention (7). As a result, social media becomes a place where empowerment and insecurity often exist side by side.

Social media's influence is not limited to patients or consumers; it also reshapes professional medical practice. Surgeons and aesthetic practitioners increasingly use digital platforms to display results, educate the public, and build their professional image. Although these efforts can democratize information, they also blur the boundary between health education and marketing (8). Ethical reviews have warned that exaggerated claims, selective image use, and absence of disclosure can mislead audiences and diminish trust (9). Practicing medicine online requires a careful balance between being visible and being responsible—maintaining honesty, accuracy, and respect for patients at all times.

One of the fields most shaped by these trends is cosmetic gynecology. It has grown rapidly in recent years while remaining ethically and culturally sensitive. In this area, female genital cosmetic surgery (FGCS) includes procedures that enhance comfort and appearance. Many women choose these treatments to feel more confident, enhance satisfaction, or relieve physical discomfort. However, the motivations behind these choices are rarely independent of cultural context or social imagery (10). In Saudi Arabia, discussions about body image and sexuality are still traditionally private. But social media has become both a space of freedom and a source of pressure. It allows women to speak more openly about their aesthetic choices, yet at the same time encourages new, digitally shaped ideas of what femininity should look like.

Empirical evidence reflects this duality. A Saudi cross-sectional study found that women who underwent FGCS were generally highly satisfied with their postoperative results, yet many acknowledged that their expectations had been shaped by visual and testimonial content encountered online (11). In this way, social media works in two directions: it helps reduce stigma around intimate procedures while also setting new benchmarks for what is considered “normal.” It opens space for discussion, yet it also encourages conformity—empowering some women while quietly influencing the motivations of others.

These changes raise important ethical and professional questions because cosmetic gynecology is involved with such personal areas of a woman's life, this requires more patient counseling, consent, and education. Doctors also need to make sure that women understand both the benefits and the limits of these procedures and that decisions should be made for personal reasons rather than social pressure. The Plastic and Reconstructive Surgery review on social media ethics highlights the importance of protecting patient privacy, being transparent about sponsorships, and avoiding exaggerated or misleading posts (8). For doctors working in culturally conservative societies, the real challenge is finding the right balance—respecting a woman's choice while helping her avoid expectations shaped by unrealistic online ideals.

Even though social media's effect on cosmetic surgery has been widely studied, very little research looks at its role in cosmetic gynecology. Most studies focus on facial or skin procedures and pay little attention to how online content shapes women's choices about more private treatments. In Saudi Arabia, the few studies that exist mostly describe how common these procedures are, but they don't explore the reasons behind them. As a result, we still don't have a clear understanding of how culture, social media, and personal motivations come together to influence women's decisions in this area.

This review aims to help close that gap. It brings together global and local studies published between 2018 and 2024 to explore how social media affects women's views, motivations, and decisions about cosmetic procedures, especially those related to gynecologic aesthetics. By looking at psychological, cultural, and ethical aspects, this paper offers a clearer picture of how social media influences both patients and practitioners in today's fast-changing world. The goal is not just to describe the trends but to guide doctors and researchers in understanding the challenges and responsibilities that come with the growing impact of digital media on aesthetic medicine.

## Methods

### Research questions

This review was guided by the following research questions:

1. How does social media exposure influence women's perceptions and decisions regarding cosmetic and gynecologic procedures?
2. What psychological mechanisms mediate this relationship?
3. What ethical and cultural considerations shape aesthetic decision-making within the Saudi context?

### Study design

This paper adopted a structured narrative review design to synthesize and interpret published evidence on the influence of social media on cosmetic and gynecologic aesthetic decisions among women. The narrative approach was selected to allow the integration of diverse findings—quantitative, qualitative, and conceptual—within a coherent discussion relevant to cultural and professional contexts in Saudi Arabia.

### Search strategy

A comprehensive search was conducted for studies published between January 2018 and March 2024 using PubMed, OpenAlex, and institutional repositories. The search combined Medical Subject Headings (MeSH) and free-text terms as follows:

(“social media” OR “advertising” OR “promotion” OR “marketing”) AND (“aesthetic medicine” OR “cosmetic surgery” OR “cosmetic gynecology” OR “female genital cosmetic surgery”) AND (“decision-making” OR “perception” OR “attitude” OR “influence” OR “ethics”).

Filters limited the results to peer-reviewed English-language publications focusing on human subjects and female populations. Additional sources were identified through manual screening of reference lists in relevant papers.

The final literature search was completed on 3 November 2025. Gray literature, including academic theses, was intentionally included to capture regional evidence not always indexed in major databases. Only English-language publications were included, which may have resulted in the exclusion of some relevant regional studies.

### Study selection

- Titles and abstracts were screened for relevance to three predefined themes:
- The influence of social media on aesthetic or cosmetic decision-making.
- The ethical and professional use of social media in medical or surgical practice.

Research addressing female genital cosmetic surgery (FGCS), cultural perspectives, or psychological motivations within Saudi or broader Middle-Eastern settings.

Duplicate records were removed, and full texts were retrieved when available. Only verified and accessible publications were included to ensure transparency and reproducibility.

Screening and study selection were performed by a single author; therefore, no inter-reviewer agreement assessment was conducted.

### Data extraction and synthesis

From each study, information was extracted on the year of publication, country or region, study type, sample size, target population, key variables (perceptions, motivations, ethical considerations), and principal findings. Reported limitations were also recorded.

### Data were organized into four thematic domains

- Behavioral influence of social media
- Psychological and motivational factors
- Ethical and professional considerations

### Review approach and appraisal

This work was designed as a structured narrative review to synthesize evidence across heterogeneous study types (cross-sectional surveys, reviews, and academic theses) and to integrate cultural and ethical dimensions that are not easily captured in quantitative synthesis. The review was guided by the SANRA principles for narrative reviews to improve transparency and structure. Formal risk of bias scoring was not performed instead, methodological strengths and limitations of included sources (study design, measures of exposure/outcomes, and sampling) are discussed narratively in the Discussion.

### Cultural context and implications for Saudi society

A thematic narrative synthesis was then applied to integrate findings across these domains. This approach enabled both statistical observations and qualitative insights to be interpreted together, highlighting links between digital exposure, personal motivation, and social perception.

### Ethical considerations

The review was conducted under ethical approval from the King Abdulaziz University Research Ethics Committee (Reference No. 144-25). All procedures adhered to the principles of the Declaration of Helsinki and institutional standards for non-interventional research.

Ethical concerns related to social media use in aesthetic medicine must be considered within existing professional and regulatory frameworks. International guidance, including medical professionalism standards and digital health ethics principles, emphasizes transparency, patient confidentiality, and the avoidance of misleading or exaggerated claims. Within this context, ethical risks differ between non-medical influencers and licensed healthcare professionals. Influencers often operate without clinical accountability, potentially promoting unrealistic expectations, whereas healthcare professionals are bound by professional codes of conduct that require balanced information, informed consent, and respect for patient dignity—responsibilities that are particularly critical in culturally conservative settings such as Saudi Arabia.

## Results

### Overview of included studies

**Figure 1:**
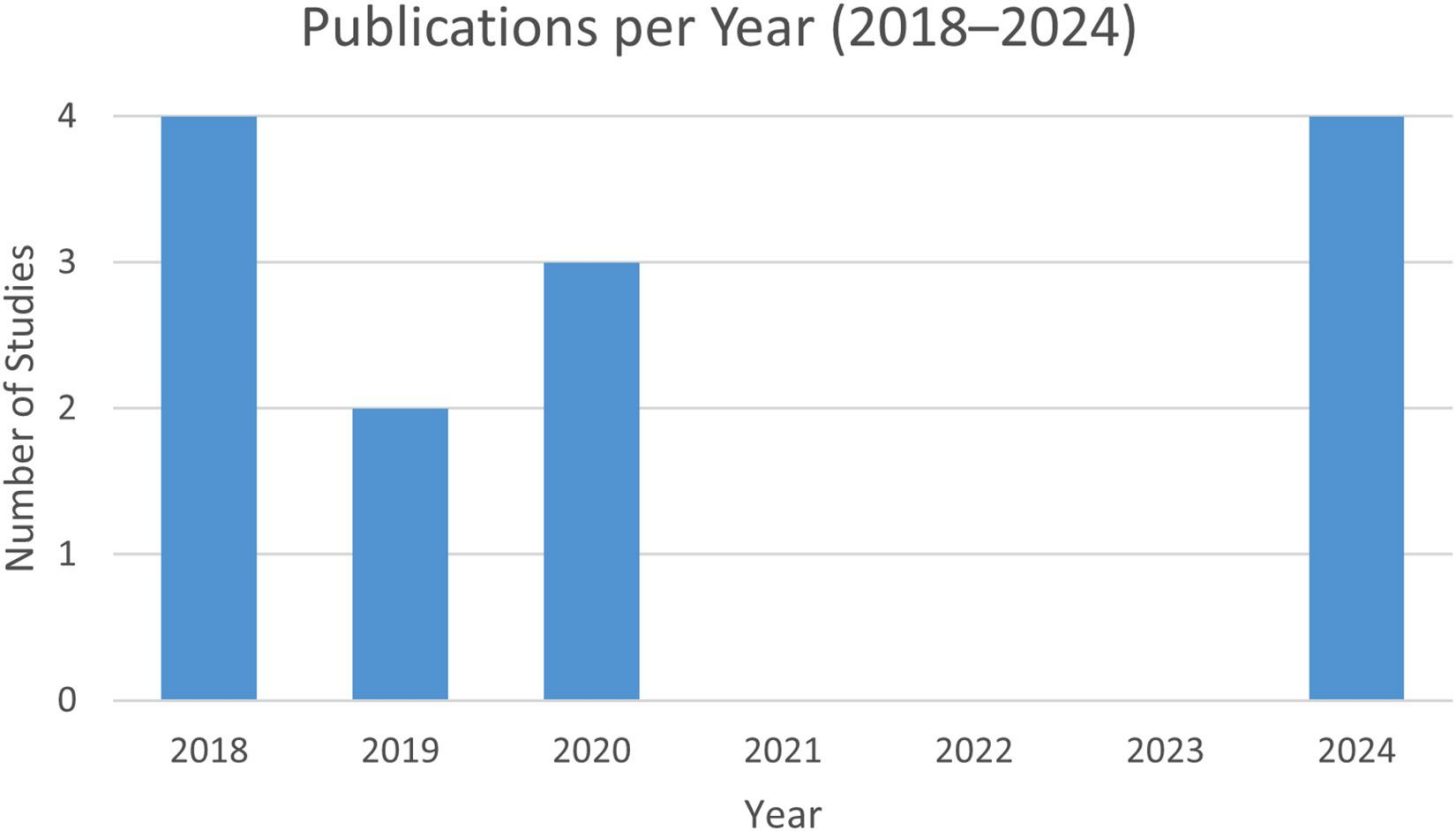
Publications per year (2018–2024) among the studies included in this review. Research activity peaked in 2018 and 2024, with no eligible studies identified between 2021 and 2023.

**Figure 2:**
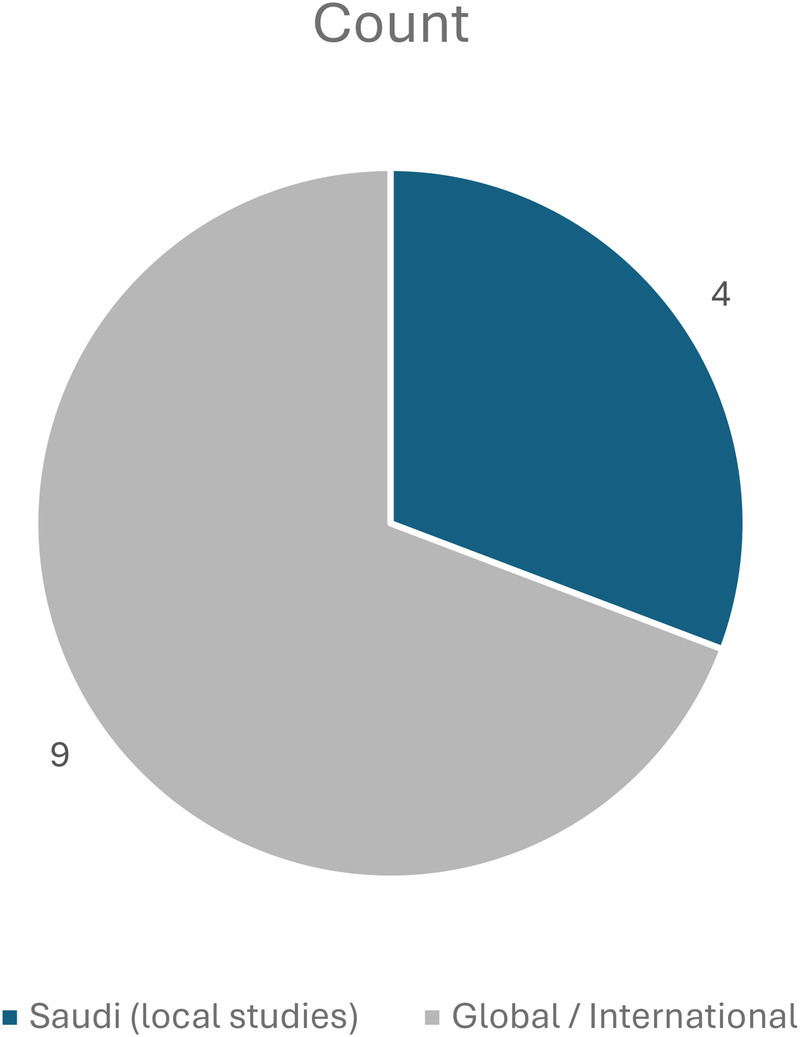
Region of evidence (Saudi vs. Global). This figure visually summarizes the main ideas that repeatedly appeared in the reviewed studies. Comparison showing four Saudi-based studies and nine international ones, highlighting the balance between local and global perspectives.

A total of 13 studies published between 2018 and 2024 were included in this review (Figure 1). The body of evidence comprised cross-sectional surveys, narrative reviews, and academic theses conducted in Saudi Arabia and other international settings such as the United States, Australia, and Europe (Figure 2). These studies explored the effects of social-media exposure, psychological outcomes, ethical concerns, and cultural influences on women's decisions regarding cosmetic and gynecologic aesthetic procedures.

Most Saudi studies conducted between 2019 and 2024 focused on the impact of Instagram and Snapchat, consistently linking high exposure to aesthetic content with increased intent to undergo cosmetic treatment. Global literature reinforced these findings by documenting body-image dissatisfaction, appearance comparison, and normalization of aesthetic modification within online environments. A condensed overview of the included studies is presented in Table 1.

**Table 1:** Summary of literature included in the narrative synthesis (2018–2024).

No.	Year	Study description	Journal/Source	Region	Theme	Key Finding (summary)
1	2024	Cross-sectional study examining social-media exposure and interest in cosmetic facial procedures	Cureus	Saudi Arabia	Decision	Instagram/Snapchat exposure associated with higher intention to consider cosmetic procedures.
2	2024	Systematic review examining the relationship between social media use, body image perception, and interest in cosmetic surgery	Cureus	Global	Psychology	Appearance-focused social media associated with body dissatisfaction and greater interest in aesthetic procedures.
3	2024	Public-health analysis discussing health and ethical risks related to beauty-product promotion on social media	Frontiers in Public Health	Global	Public Health	Highlights potential health risks and ethical concerns related to online beauty trends.
4	2020	Cross-sectional study assessing satisfaction following female genital cosmetic procedures	Aesthetic Surgery Journal Open Forum	Saudi Arabia	FGCS	High postoperative satisfaction reported; motivations partly influenced by online information.
5	2019	Survey study investigating the influence of social media on cosmetic-procedure decision-making among Saudi women	PRS Global Open	Saudi Arabia	Decision	Approximately half of participants reported social media influence on cosmetic-procedure consideration.
6	2019	Cross-sectional survey examining social-media impact on attitudes toward aesthetic procedures	Cureus	Saudi Arabia	Decision	Filters, influencer posts, and before-after images associated with increased procedural interest.
7	2020	Review discussing professional use of social media in plastic-surgery practice	Aesthetic Plastic Surgery	Global	Ethics	Highlights benefits of digital engagement while emphasizing professionalism and ethical responsibility.
8	2018	Systematic review examining ethical issues related to plastic-surgery content shared on social media	Plastic and Reconstructive Surgery	Global	Ethics	Emphasizes transparency, confidentiality, and responsible communication online.
9	2020	Academic thesis examining social media exposure and body-image perceptions among young women	University thesis	Australia	Psychology	Findings suggest body-positive content may still reinforce appearance-focused evaluation.
10	2018	Doctoral dissertation examining body-image constructs and attitudes toward cosmetic surgery	Doctoral dissertation	United States	Psychology	Social comparison and appearance dissatisfaction associated with increased acceptance of cosmetic surgery.
11	2018	Global cosmetic-surgery statistics report from a professional aesthetic-surgery society	Professional society report	Global	Trends	Reports rising global demand for cosmetic procedures among female patients.
12	2018	Conceptual article discussing definitions and scope of cosmetic vs. reconstructive surgery	Australasian Journal of Plastic Surgery	Global	Definition	Clarifies distinctions between cosmetic and reconstructive procedures.
13	2024	Cross-cultural analysis examining how beauty advertising shapes aesthetic ideals across societies	Open-access journal article	Global	Culture	Beauty ideals vary across cultures and influence perceptions of attractiveness.

**Caption:** Overview of literature included in this narrative synthesis, summarizing study type, thematic focus, and key findings related to social-media influence on cosmetic and gynecologic aesthetic decision-making.

### Behavioral influence of social media

Across the literature, visual-platform engagement—particularly through influencer testimonials, filters, and before-and-after imagery—was strongly associated with greater willingness to pursue cosmetic procedures. Surveys among Saudi women showed that nearly half acknowledged direct influence from social-media exposure on their treatment considerations.

Data displayed in Graph 1 illustrate this behavioral trend: individuals with high daily exposure to aesthetic content were substantially more likely to express intent to consult a practitioner compared with those with limited exposure. Repeated online visibility and social endorsement thus appear to serve as subtle behavioral drivers normalizing cosmetic procedures.

### Psychological and motivational factors

**Figure 3:**
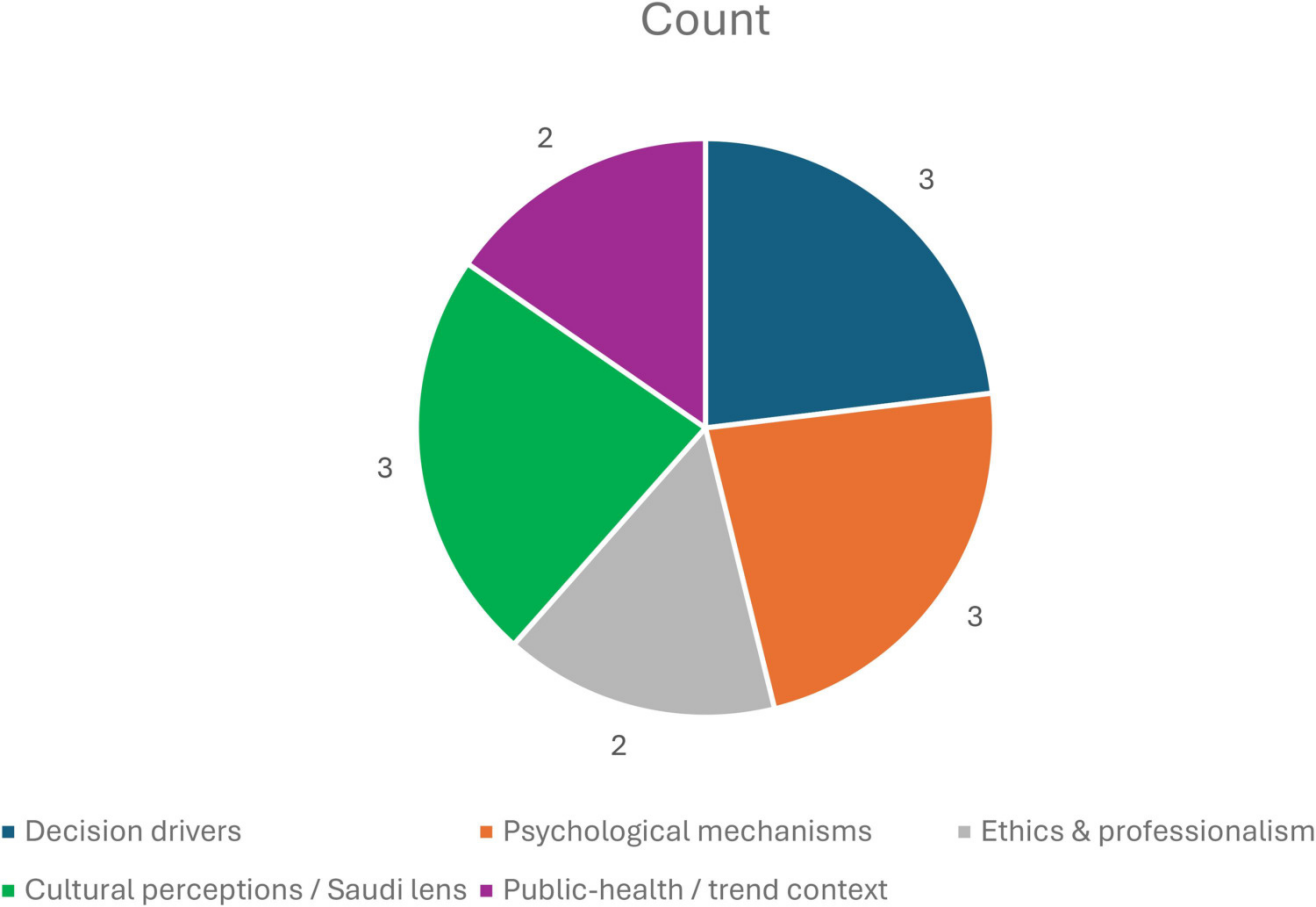
Distribution of included studies by primary theme. This figure visually summarizes the main ideas that repeatedly appeared in the reviewed studies. Proportion of studies addressing decision drivers, psychological mechanisms, ethical practice, cultural aspects, and public-health trends.

Social-media use also activates several psychological pathways, including social comparison, appearance anxiety, and self-objectification. Multiple studies observed that even body-positive content, while intended to promote acceptance, often reinforces appearance-focused norms. In Saudi cohorts, low self-esteem and peer validation emerged as major motivators, with aesthetic procedures perceived less as vanity and more as self-confidence enhancement. The thematic distribution of the included literature across these domains is summarized in Figure 3.

As shown in Graph 2, increased exposure to influencer content corresponded with higher body-image concern scores, highlighting how digital environments shape motivation and self-perception. Together, these findings suggest a cyclical pattern in which visibility and validation perpetuate aesthetic desire.

### Ethical and professional considerations

Ethical analysis featured prominently in professional publications. Plastic-surgery and aesthetic-medicine journals emphasized that online visibility, while beneficial for education and outreach, may blur the distinction between patient education and marketing. Reported concerns included exaggerated results, selective use of imagery, and lack of sponsorship disclosure.

Saudi clinicians were encouraged to adhere strictly to national and international ethical codes governing digital communication and to maintain transparency and confidentiality in their online presence. Graph 3 summarizes current trends in practitioner awareness, indicating generally positive attitudes toward ethical responsibility but inconsistent application in practice.

### Cultural context and Saudi perspective

The Saudi context remains pivotal for understanding the influence of social media on aesthetic decision-making. Rapid social change, evolving norms, and strong digital engagement create a complex environment where empowerment coexists with subtle social pressure. Local research showed that aesthetic motivation often reflects both cultural expectations and digital imagery, rather than purely personal preference.

Studies on female genital cosmetic surgery (FGCS) revealed high satisfaction with postoperative results but noted that expectations were frequently shaped by online testimonials and influencer narratives. Table 2 provides a thematic synthesis of these cross-cultural and ethical dimensions, demonstrating how Saudi women interpret social-media messages through the lens of cultural values and modesty norms.

**Table 2:** Thematic synthesis of evidence from included studies.

Theme	Evidence summary (paraphrased)	Representative sources
Decision drivers (exposure intention)	Influencer/testimonial content, filters, and before–after imagery normalize procedures and increase perceived need and willingness to consider treatment.	PRS Global Open 2019; Cureus 2019 (Riyadh); Cureus 2024 (Saudi)
Psychological mechanisms	Social comparison, appearance anxiety, and self-objectification mediate the link between platform exposure and cosmetic-surgery interest; “body-positive” streams can still be appearance-centric.	Cureus 2024 SR; UTS thesis 2020; dissertation 2018
Ethics & professionalism online	Social media supports education and practice growth but invites risks: exaggerated claims, inadequate risk framing, confidentiality concerns, conflicts of interest.	Aesthetic Plastic Surgery 2020; PRS 2018 ethics SR
Cultural/Saudi lens	High visual-platform use intersects with evolving norms; Saudi surveys show strong platform effects on perceptions and intent.	Cureus 2019 & 2024 (Saudi); ASJ Open Forum 2020; Cross-cultural ads 2024
Public-health & trends	Rising global demand and beauty-norm pressures influence decision-making; product risk discourse is often overshadowed by promotional content.	ASAPS 2018 statistics; Frontiers in Public Health 2024

Synthesis of key evidence grouped into five major themes: decision drivers, psychological mechanisms, ethics and professionalism, cultural/Saudi context, and public-health trends.

### Integrated conceptual model

**Figure 4:**
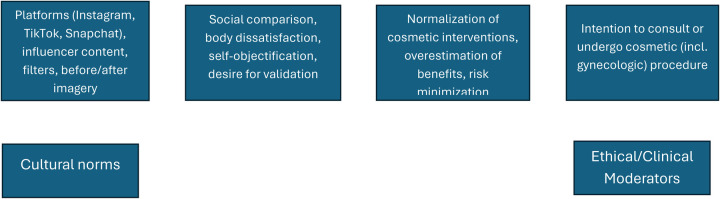
Conceptual framework linking social media exposure to cosmetic-procedure decisions. This figure visually summarizes the main ideas that repeatedly appeared in the reviewed studies. Exposure to influencer content and aesthetic imagery initiates psychological processes—social comparison, body dissatisfaction, and objectification—that reshape perceptions of beauty and normalize aesthetic interventions. These processes, moderated by cultural and ethical factors, influence behavioral decisions to pursue cosmetic (including gynecologic) procedures.

The relationship between exposure, psychology, and behavior is illustrated in Figure 4, which presents a conceptual framework linking social-media engagement to decision-making about aesthetic procedures. Exposure to curated imagery and influencer content initiates psychological responses—social comparison, body dissatisfaction, and self-objectification—that alter perceptions of beauty. These processes, moderated by cultural and ethical boundaries, influence behavioral intent toward cosmetic and gynecologic enhancement.

### Practical recommendations and future directions

Table 3 outlines practical recommendations and research priorities emerging from the review. Clinicians are encouraged to address social-media influence explicitly during patient counseling and informed-consent discussions, promoting realistic expectations and media literacy. Ethical guidance should emphasize transparent advertising and avoidance of misleading visuals or undisclosed sponsorships.

**Table 3:** Practical recommendations and research implications derived from the review.

Focus area	Practical recommendations	Research implications
Clinical counseling	Include discussion of social-media influence when taking patient history and during pre-procedure counseling. Use media-literacy approaches to correct unrealistic expectations.	Study effectiveness of educational interventions or counseling programs that address social-media influence on patient decisions.
Professional ethics & regulation	Adhere to national and international guidelines for professional use of social media; avoid misleading before/after imagery and undisclosed sponsorships.	Examine compliance with ethical advertising standards among clinics in Saudi Arabia and the Gulf region.
Public education	Promote campaigns that emphasize authentic body image and informed decision-making about aesthetic procedures.	Evaluate public-health impact of social-media awareness campaigns on cosmetic-procedure attitudes.
Cultural context	Tailor patient education to align with Saudi social norms and religious sensitivities regarding modesty and self-image.	Conduct qualitative research exploring cultural perceptions of aesthetic modification and media influence.
Future research directions	Encourage longitudinal Saudi studies linking quantified exposure to validated psychological scales.	Develop predictive models integrating psychological, social-media, and cultural variables influencing cosmetic-procedure uptake.

Summary of proposed clinical, ethical, and research actions for healthcare professionals and investigators addressing social-media impact on aesthetic and gynecologic procedures.

Future research should focus on longitudinal Saudi studies integrating psychological scales with measured exposure data and on developing predictive models that incorporate social, ethical, and cultural variables influencing women's cosmetic decisions.

## Discussion

This review shows how social media has become one of the strongest influences on women's ideas about beauty and their choices about cosmetic procedures. In Saudi Arabia, where digital life and social change are moving quickly together, social media often serves as both a teacher and a mirror. It teaches new standards of beauty while reflecting what people believe others expect from them. Similar patterns have been described in other countries, where social media encourages constant self-comparison and turns appearance into a form of communication and identity (1, 2).

### The power of online images

Across most of the studies reviewed, women who spent more time on visual platforms like Instagram, Snapchat, or TikTok were more likely to say they were thinking about or planning a cosmetic procedure (3, 4). What seems to drive this is not only the content itself, but also the repetition and the social approval it brings. Each “like,” comment, or influencer post quietly reinforces the message that looking better can mean feeling better or being more accepted. The more people see such images, the more normal cosmetic change appears (5).

### How social Media affects feelings and motivation

Beyond behavior, social media also shapes how people see and feel about themselves. Constant exposure to flawless or filtered images invites comparison, often leading to self-doubt and body dissatisfaction (6). Even pages that claim to promote body positivity can, unintentionally, keep the focus on appearance rather than acceptance (12). Among Saudi women, cosmetic procedures were frequently described as a way to feel more confident or to align with modern beauty ideals, rather than a pursuit of vanity (4). This shows that social media can be both reassuring and demanding—encouraging self-expression while quietly measuring it against idealized standards. These observations are supported by evidence showing that lower self-esteem, reduced life satisfaction, and heightened concerns about physical attractiveness are significantly associated with greater acceptance of cosmetic surgery, underscoring the role of psychological factors in aesthetic decision-making (7).

### Professional and ethical concerns

As social media becomes an essential part of how medicine is presented to the public, new questions are emerging about ethics and professionalism. Many studies in aesthetic and plastic surgery warn that what appears online is not always the full story—results can be overstated, and risks quietly left out (8, 9). For doctors and clinics in Saudi Arabia, the challenge is even more delicate: being visible and approachable online while still respecting the country's strong values of modesty and privacy. Good ethics online start with honesty, clear consent, and being upfront about what's shown or sponsored. When doctors are trained to follow these simple principles, they can communicate openly and confidently without losing the trust that makes their work meaningful.

### Culture and context

Culture plays a big part in how women decide about their appearance. In Saudi Arabia, many women try to balance long-held traditions with the new freedom that social media brings (10). For many, cosmetic choices sit somewhere in between—a way to feel confident and modern while still staying true to their cultural and religious values. In studies on female genital cosmetic surgery (FGCS), most women said they were happy with their results, but many also admitted that social media had shaped their ideas of what is “normal” or “beautiful.” (11) It shows how culture and online influence often come together, shaping choices that feel personal but are also part of a shared social change.

### Seeing the bigger picture

The model in Figure 4 helps explain how this process develops. Exposure to idealized online images encourages comparison and self-criticism, which slowly reshapes how people see themselves and what they consider attractive. Over time, this creates a continuous cycle in which social media doesn't just reflect beauty ideals—it defines them. Understanding this pattern makes it clear that awareness and open discussion are key to helping women make choices that are confident, informed, and truly their own. When ethical awareness and cultural understanding are part of the conversation, that influence can be softened, giving women space to make choices that feel informed and genuinely their own (13).

### Practical lessons and future directions

Doctors and counselors should recognize that almost every patient today arrives with ideas shaped by what they have seen online. Discussing social media influence openly—especially during counseling—can help patients set realistic expectations and feel more empowered in their decisions. Educational programs and media-literacy campaigns may also help women understand how filters, lighting, and digital editing affect what they see.

For researchers, the next step in Saudi Arabia is to look at how ongoing exposure to social media shapes body image and decision-making over time. Following people's experiences instead of taking a single snapshot could reveal much more about how digital culture affects real choices. Using psychological tools alongside measures of media use would help paint a clearer picture of how online influence translates into everyday behavior (14).

While the reviewed studies consistently report an association between social media exposure and interest in cosmetic procedures, there are notable differences in study design and evidence strength. Most available evidence is derived from cross-sectional surveys, which are useful for identifying patterns but cannot establish causality. In contrast, review articles and academic theses provide broader contextual insights but rely on heterogeneous methodologies. Measures of social media exposure also varied considerably across studies, ranging from time spent on platforms to perceived influence of specific content or influencers, which limits direct comparison of findings. Similarly, decision-making outcomes were inconsistently defined, spanning intention, consideration, and actual procedure uptake. The predominance of cross-sectional designs and self-reported measures highlights the need for more longitudinal and experimental research to better understand causal pathways.

## Limitations

This review has several limitations that should be considered when interpreting the findings. First, the available literature is dominated by cross-sectional studies, which limits the ability to draw causal conclusions regarding the influence of social media on cosmetic decision-making. Second, most studies rely on self-reported data, increasing the potential for recall and social desirability bias. Third, there is a possibility of publication bias, as studies reporting positive associations may be more likely to be published. Additionally, the findings may not be fully generalizable beyond digitally active female populations, and the rapidly evolving nature of social media platforms may limit the long-term applicability of some conclusions. Finally, the absence of longitudinal or experimental studies restricts insight into changes over time and causal pathways.

## Conclusion

Social media has become a part of everyday life, shaping how women see themselves and what they believe is beautiful. It can be inspiring—giving women the chance to express confidence and individuality—but it can also bring quiet pressure to look a certain way. In Saudi Arabia, these influences meet a rich cultural backdrop, where tradition and modern life often move side by side.

For doctors and others working in this field, finding that balance really matters. Talking openly with patients about how social media shapes what they see and expect can help them make choices that truly feel right for them not choices influenced by comparison or pressure.

In conclusion, we need to conduct more studies that listen to what women want to say and follow their experiences over time. This will allow the medical community can better support women in making decisions that come from confidence and genuine self-understanding.

The relatively limited number of included studies reflects the emerging nature of research specifically addressing social media influence on cosmetic and gynecologic decision-making within Saudi and comparable cultural contexts.

As screening was conducted by a single reviewer, the possibility of selection bias cannot be entirely excluded.
